# NCOurd: modelling length distributions of NCO events and gene conversion tracts

**DOI:** 10.1093/bioinformatics/btad485

**Published:** 2023-08-03

**Authors:** Marteinn T Hardarson, Gunnar Palsson, Bjarni V Halldorsson

**Affiliations:** deCODE genetics, Reykjavik 102, Iceland; School of Technology, Reykjavik University, Reykjavik 102, Iceland; deCODE genetics, Reykjavik 102, Iceland; deCODE genetics, Reykjavik 102, Iceland; School of Technology, Reykjavik University, Reykjavik 102, Iceland

## Abstract

**Motivation:**

Meiotic recombination is the main driving force of human genetic diversity, along with mutations. Recombinations split into crossovers, separating large chromosomal regions originating from different homologous chromosomes, and non-crossovers (NCOs), where a small segment from one chromosome is embedded in a region originating from the homologous chromosome. NCOs are much less studied than mutations and crossovers as NCOs are short and can only be detected at markers heterozygous in the transmitting parent, leaving most of them undetectable.

**Results:**

The detectable NCOs, known as gene conversions, hide information about NCOs, including their number and length, waiting to be unveiled. We introduce NCOurd, software, and algorithm, based on an expectation–maximization algorithm, to estimate the number of NCOs and their length distribution from gene conversion data.

**Availability and implementation:**

https://github.com/DecodeGenetics/NCOurd

## 1 Introduction

The name NCOurd (pronounced encored) is a combination of NCO and Urd. In Nordic mythology, Urd is one of the three Norns (witches) who decide the fate of men and gods. Of the three witches, Urd represents the past, and we would love to understand NCOs happening in the past, during meiosis, but we can only measure gene conversion in the present, from the DNA of living individuals. With mathematical (mathe-magical) witchery, we are able to estimate the past from the present.

In sexually reproducing organisms, the gametes (reproductive cells) are created in a special cell division called meiosis. Meioses are initiated by diploid cells, but the product, the gametes, are haploid and each of their chromosomes is a combination of the two homologous chromosomes of the parent cell created by recombinations. Recombinations allow offspring to inherit a mosaic of the homologous parental chromosomes rather than a copy of either one of them.

Determining from which homologous parental chromosome a segment originates is only possible at heterozygous markers; sites where the two chromosomes differ. Meiotic recombinations are initiated by a double strand break (DSB) in one parental chromosome and are repaired using the homologue as a template. This can result in either a crossover, which separates large chromosomal segments from different homologues, or non-crossover (NCO). DSBs that are repaired with NCOs can result in gene conversions where alleles from one haplotype are embedded on the background of the homologous haplotype. The gene-converted markers from a single NCO form a gene conversion tract. (See Supplementary Fig. S1 and Lam and Keeney (2014) for more information on the meiotic recombination process.)

**Figure 1.**
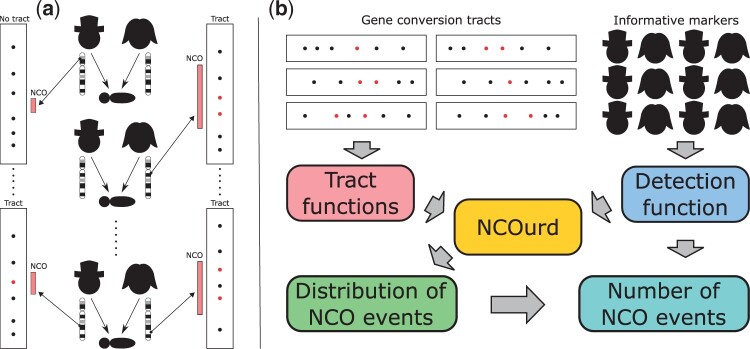
(a) NCO events (red rectangles) occur during meiosis. Some NCO events overlap heterozygous markers (points) and create gene conversion (red points), alleles from one haplotype embedded in the background of the homologous haplotype (black points). If at least one marker gets gene converted a gene conversion tract is created. (Note that gene conversion tracts do not need to consist of consecutive heterozygous markers.) The penetrance is probability of a heterozygous marker overlapping the NCO event becoming a gene conversion. (b) For each observed gene conversion tract, a tract function is computed, representing the probability that the gene conversion tract was produced by a NCO event of length *x*. A detection function, representing the probability that a gene conversion can be detected from a NCO event of length *x*, is calculated based on the all heterozygous markers in all the parents. NCOurd uses these functions to estimate the length distribution and the number of NCO events.

Not all heterozygous markers overlapping an NCO event will become gene conversions (Supplementary Fig. S1) so NCO events can extend beyond the heterozygous markers flanking the gene conversion tract and the gene conversion tract does not need to consist of consecutive heterozygous markers. Some NCO events will fail to result in a gene conversion tract either because the NCO event did not overlap any heterozygous markers or because none was gene converted (Fig. 1a).

Consequently, short NCO events are very unlikely to produce any gene conversion, but as events get larger, the probability increases, upwardly skewing the length distribution of NCO events leading to gene conversions compared with the underlying length distribution of all NCO events. Furthermore, looking at individual gene conversions gives limited insights into the length of the original NCO event as flanking heterozygous markers can be far away or may have overlapped the NCO event but not been gene converted. These two factors make it impossible to estimate the length distribution of NCO events without some statistical modelling.

The scope of this article is limited to directly observable allelic gene conversion, which have been studied in various species. For example, Williams *et al.* (2015) and Halldorsson *et al.* (2016) used three-generation families to study gene conversions in humans, Miller *et al.* (2016) studied gene conversions in fruit flies, Li *et al.* (2019) in mice, and Wall *et al.* (2022) in baboons.

Gene conversions have also been studied with population-based methods (Betrán *et al.* 1997, Setter *et al.* 2022). Non-allelic gene conversion, where a different locus is used as a template for DNA repair, has also been studied (Mansai and Innan 2010). Both settings require statistical models different from the one presented here.

Some studies attempt to estimate length distributions and the number of NCO events (Mansai *et al.* 2011, Miller *et al.* 2012, Li *et al.* 2019). Until recently, the models used have been limited to a single exponential distribution or their discrete analogue, geometric distribution. The authors of Wall *et al.* (2022) found that a single geometric distribution did not fit their data, consisting of gene conversions in baboons, well and proposed a mixture of two geometric distributions. Modelling gene conversions with a mixture distribution is reasonable, as DSBs can be resolved with an NCO via multiple pathways. However, a mixture of geometric distributions has the drawback of forcing NCO events to become increasingly more likely as they get smaller, neglecting the possibility that the strand invasion process might enforce a minimum NCO event size. Further, the models used thus far are oblivious to the fact that some heterozygous markers overlapping NCOs fail to produce gene conversions allowing NCO events the possibility to extend beyond flanking heterozygous markers of the gene conversion tract.

To improve upon this and to utilize ever larger gene conversion datasets, we introduce NCOurd, a software package to infer the length distributions of NCO events (Fig. 1b). We demonstrate the robustness of our method using simulations and apply it to published datasets (Halldorsson *et al.* 2016, Li *et al.* 2019).

Given a set of gene conversions and the length distribution of NCOs, we can estimate the number of NCOs occurring per meiosis and better understand their underlying biology and how they shape human diversity and the human evolutionary process in general. Further, reliable length estimates allow us to estimate which regions of the human genome are most affected by NCOs, whether they affect disease, fertility, or other human phenotypes, and how NCOs are affected by human genetics and environmental conditions such as parental age and sex.

## 2 Methods

Our goal is to estimate the length distribution of NCO events from a set of gene conversion tracts together with the set of heterozygous markers in the transmitting parent where gene conversions would be detectable. We assume gene conversions have already been identified and grouped into gene conversion tracts. Examples of how gene conversions can be identified can be found in Halldorsson *et al.* (2016) and Li *et al.* (2019).

We first define terminology to model NCO events and their resulting gene conversion tracts. Then, we show how a likelihood function can be written in terms of the length distribution of NCO events. This allows for a maximum-likelihood estimate of the observed gene conversion tract over the parameter space of the length distribution of NCO events, assuming that the length distribution of NCOs follows a mixture of negative binomial distributions. We then use an expectation–maximization (EM) algorithm (Dempster *et al.* 1977) to solve the maximum-likelihood problem.

### 2.1 Definitions and model

NCO events can lead to ‘gene conversions’; where one or more alleles from one chromosome are embedded in a haplotype originating from the homologous chromosome, the ‘background haplotype’. ‘Informative markers’ are all sites where a gene conversion can be detected and, thus, are a subset of the sites where a gene conversion can occur, i.e. the heterozygous markers in the parent (some heterozygous markers may be filtered for quality control). A ‘gene conversion tract’ is the set of all the gene-converted markers detected from a single NCO event. Note that a gene conversion tract need not be consecutive informative markers as some informative markers within an NCO event can be from the background haplotype. To account for the fact that not all heterozygous markers result in a gene conversion, we define the ‘penetrance’ *p* as the proportion of informative markers that are gene converted within NCO events. (Informative markers overlapping the NCO event have the probability 1−p of being reverted back to the background state).

Let (L,M,C,O) be a random NCO event where *L* is the length of the event, *M* is the starting point (lowest chromosomal coordinate), *C* is the chromosome, and O=(R,S) is the observed gene conversion tract, where *R* denotes a parent–offspring pair in which the event occurred and *S* is the set of gene-converted markers produced by the event (*S* may be empty). Both *L* and *M* are discrete random variables taking integer values, *C* and *R* are random categorical values, and *S* is a random set of integers. We also define *I_R_* as the informative markers of the parent in *R*.

The set *S* consists of zero or more informative markers on chromosome, *C*, of the parent in *R* overlapping the NCO tract, i.e. $S\subseteq [M,M+L-1]\cap I_{R}$. The random NCO event produces a gene conversion tract if and only if S≠Ø.

Let *t* be a gene conversion tract. Let $o_{t}=(r_{t},s_{t})$ be the pair consisting of *r_t_*, the transmitting parent–recipient offspring pair, and *s_t_*, the set of gene-converted markers in the tract *t*.

As an input for our EM algorithm, we need the following:

- *Detection function*, *D*(*x*), for an integer *x* is the probability of a random NCO event of length *x* producing a gene conversion tract, i.e. D(x)=Pr(S≠Ø | L=x).
  The detection function measures the probability of an NCO event of length *x* is detected (as a gene conversion) and depends on the distribution of informative markers across the genome and the penetrance.
- *Tract functions*, $T_{t}(x)$, for an integer *x* and a gene conversion tract *t* are the probability of a random NCO event of length *x* producing the gene conversion tract *o_t_*, i.e. $T_{t}(x)=Pr(O=o_{t}\;|\;L=x)$.
  The tract functions measure the probability of an NCO event of length *x* producing a tract *t*. The probabilities depend on the placement of informative markers around the gene conversion tract *t* and the penetrance.

We note that these functions can be calculated without knowledge of the length distribution of NCO events.

We need the penetrance *p* for these calculations. All informative markers within all NCO events are assumed to have a probability *p* of becoming gene conversions independently of each other. The probability of an NCO event resulting in a specific gene conversion tract contained within the NCO event is, therefore, the product of *p* and (1−p) for each informative marker within the NCO event depending on whether it was a gene conversion or not and if the NCO event has *n* informative markers the probability of any gene conversion being created is $1-{\left(1-p\right)}^{n}$ (i.e. all cases except when all informative markers inside the NCO event are reverted to the original state).

The value of the detection function at *x* is the average probability that an NCO event of length *x* creates any gene conversion tract over all possible placements in the genome.

For each gene conversion tract *t*, the value of $T_{t}(x)$ is the sum of the probabilities of an NCO event of length *x* resulting in *t* over all possible placements containing *t*, weighted with the probabilities of the placement.

The penetrance can be estimated by computing the fraction of gene-converted markers in all gene conversion tracts, excluding boundary markers (the first and last marker of each tract). It is possible that the penetrance depends on the NCO event length (i.e. short tracts could be either more or less likely to produce gene-converted markers). The calculations of detection and tract functions can easily be augmented to accommodate penetrance as a function of NCO event length. However, estimating penetrance as a function of NCO event length is difficult and would likely suffer from lack of power.

A more detailed description of how the detection and the tract functions are calculated and the approximations used together with information on how the penetrance can be estimated can be found in the Supplementary Information.

### 2.2 Likelihood function

To calculate a likelihood function, we must be able to evaluate the probability of our outcomes. Using our model, we assume random NCO events are generated, but we can only observe them if they create a gene conversion tract (S≠Ø). In other words, for gene conversion tract *t*, we need to estimate


$$Pr(O=o_{t}|S\neq Ø).$$

Using the law of total probability, we get:


(1) $$Pr(O=o_{t}|S\neq Ø)=\sum_{x=1}^{\infty }Pr(O=o_{t}|S\neq Ø,L=x)\cdot Pr(L=x|S\neq Ø).$$

The two factors inside the sum can be rewritten using our definitions of detection and tract functions. The former, together with the definition of conditional probability, gives: since *t* is a gene conversion tract, *O* = *o_t_* implies that S≠Ø. The latter combined with the Bayes’ theorem and the law of total probability giving:


(2) $$\begin{array}{c} Pr(O=o_{t}|S\neq Ø,L=x)=\frac{Pr(O=o_{t},S\neq Ø|L=x)}{Pr(S\neq Ø|L=x)}\\ =\frac{Pr(O=o_{t}|L=x)}{Pr(S\neq Ø|L=x)}=\frac{T_{t}(x)}{D(x)}, \end{array}$$

(3) $$\begin{array}{c} Pr(L=x|S\neq Ø)=\frac{Pr(S\neq Ø|L=x)\cdot Pr(L=x)}{Pr(S\neq Ø)}\\ =\frac{Pr(S\neq Ø|L=x)\cdot Pr(L=x)}{\sum_{y=1}^{\infty }Pr(S\neq Ø|L=y)\cdot Pr(L=y)}\\ =\frac{D(x)\cdot Pr(L=x)}{\sum_{y=1}^{\infty }D(y)\cdot Pr(L=y)}. \end{array}$$

Putting the three equations together (1)–(3), we get:


(4) Pr(O=ot|S≠Ø)=∑x=1∞Pr(O=ot|S≠Ø,L=x)·Pr(L=x|S≠Ø)=∑x=1∞Tt(x)D(x)·D(x)·Pr(L=x)∑y=1∞D(y)·Pr(L=y)=∑x=1∞Tt(x)·Pr(L=x)∑x=1∞D(x)·Pr(L=x).

We are now ready to define our likelihood function. We assume that we have a set of gene conversion tracts, T, and *L* is a discrete parametric distribution with parameters *θ*, denoted $L_{\theta }$.

Then we want find *θ* which maximizes the likelihood function the second equation is due to Equation (4).


(5) $$L(\theta |T)=\prod_{t\in T}Pr(O=o_{t}|\theta ,S\neq Ø)=\prod_{t\in T}\frac{\sum_{x=1}^{\infty }T_{t}(x)\cdot Pr(L_{\theta }=x)}{\sum_{x=1}^{\infty }D(x)\cdot Pr(L_{\theta }=x)},$$

Note that even though the gene conversion tracts are drawn from a subset of all NCO events, namely requiring S≠Ø, we have written the likelihood functions in terms of length distribution of all NCO events with $Pr(L_{\theta }=x)$ together with the detection function and the tract functions assumed to have already been calculated.

### 2.3 EM algorithm

In the Supplementary Information, we show that if the length distribution of NCO events is a mixture distribution with *n* components, then the length distribution of gene conversion producing NCO events is a mixture distribution with *n* components, and the probability mass function (PMF) of each component in the second mixture distribution can be derived from the PMF of the corresponding component in the first mixture distribution. Also, if the PMFs and mixture weights for all the components are known for one of the mixture distributions, we can calculate all the weights for the other mixture distribution.

We have assumed that the length distribution of NCO events is a mixture of *n* components and let *α_i_* and ${\hat{\alpha }}_{i}$ be the mixture weights for component *i* in the length distributions of NCO events and gene conversion producing NCO events, respectively. Similarly, let *f_i_* and ${\hat{f}}_{i}$ be the PMFs for component *i* in the length distributions of NCO events and gene conversion producing NCO events, respectively. We add to our model a latent random integer, *Z*, representing membership in the mixture components. Now we have:


$$\begin{array}{c} \alpha_{i}=Pr(Z=i)\\ f_{i}(x)=Pr(L=x|Z=i)\\ f(x)=Pr(L=x)=\sum_{i=1}^{n}\alpha_{i}f_{i}(x)\\ {\hat{\alpha }}_{i}=Pr(Z=i|S\neq Ø)\\ {\hat{f}}_{i}(x)=Pr(L=x|Z=i,S\neq Ø)\\ \hat{f}(x)=Pr(L=x|S\neq Ø)=\sum_{i=1}^{n}{\hat{\alpha }}_{i}{\hat{f}}_{i}(x). \end{array}$$

We assume that the mixture components are negative binomial distributions having parameters *θ_i_* for the *i*th component and write $f_{\theta_{i}}$ instead of *f_i_*. The NCO events associated with our gene conversion tracts are from the length distribution having the PMF: and we want to determine the parameters $\hat{\alpha }=({\hat{\alpha }}_{1},\ldots ,{\hat{\alpha }}_{n})$ and $\theta =(\theta_{1},\ldots ,\theta_{n})$ (the parameters of the underlying negative binomial distributions of the NCO length distribution mixture).


$$\hat{f}(x)=\sum_{i=1}^{n}{\hat{\alpha }}_{i}{\hat{f}}_{\theta_{i}}(x)$$

Using Equation (5), our likelihood function for a single tract, *t*, now becomes: and the complete likelihood function is then:


$$\begin{array}{c} L(\hat{\alpha },\theta |t)=\frac{\sum_{x=1}^{\infty }T_{t}(x)\cdot Pr(L_{\hat{\alpha },\theta }=x)}{\sum_{x=1}^{\infty }D(x)\cdot Pr(L_{\hat{\alpha },\theta }=x)}\\ =\sum_{i=1}^{n}Pr(Z=i|S\neq Ø)\frac{\sum_{x=1}^{\infty }T_{t}(x)\cdot Pr(L_{\hat{\alpha },\theta }=x|Z=i)}{\sum_{x=1}^{\infty }D(x)\cdot Pr(L_{\hat{\alpha },\theta }=x|Z=i)}\\ =\sum_{i=1}^{n}{\hat{\alpha }}_{i}\frac{\sum_{x=1}^{\infty }T_{t}(x)\cdot Pr(L_{\theta_{i}}=x)}{\sum_{x=1}^{\infty }D(x)\cdot Pr(L_{\theta_{i}}=x)}\\ =\sum_{i=1}^{n}{\hat{\alpha }}_{i}\frac{\sum_{x=1}^{\infty }T_{t}(x)\cdot f_{\theta_{i}}(x)}{\sum_{x=1}^{\infty }D(x)\cdot f_{\theta_{i}}(x)}. \end{array}$$

(6) $$L(\hat{\alpha },\theta |T)=\prod_{t\in T}\sum_{i=1}^{n}{\hat{\alpha }}_{i}\frac{\sum_{x=1}^{\infty }T_{t}(x)\cdot f_{\theta_{i}}(x)}{\sum_{x=1}^{\infty }D(x)\cdot f_{\theta_{i}}(x)}.$$

We can now use an EM algorithm to estimate the parameters $\hat{\alpha }$ and θ that maximize the likelihood function. Assume that at step *k* in the EM algorithm, the estimated values of $\hat{\alpha }$ and θ are ${\hat{\alpha }}^{\left(k\right)}=({\hat{\alpha }}_{1}^{\left(k\right)},\ldots ,{\hat{\alpha }}_{n}^{\left(k\right)})$ and $\theta^{\left(k\right)}=(\theta_{1}^{\left(k\right)},\ldots ,\theta_{n}^{\left(k\right)})$, respectively. For a gene conversion tract *t*, we get the *i*th membership weight (E-step):


(7) $$w_{i}^{t}=\frac{{\hat{\alpha }}_{i}Pr(O=o_{t}|\theta_{i},S\neq Ø)}{\sum_{j=1}^{n}{\hat{\alpha }}_{j}Pr(O=o_{t}|\theta_{j},S\neq Ø)}=\frac{{\hat{\alpha }}_{i}\frac{\sum_{x=1}^{\infty }T_{t}(x)f_{\theta_{i}}(x)}{\sum_{x=1}^{\infty }D(x)f_{\theta_{i}}(x)}}{\sum_{j=1}^{n}{\hat{\alpha }}_{j}\frac{\sum_{x=1}^{\infty }T_{t}(x)f_{\theta_{j}}(x)}{\sum_{x=1}^{\infty }D(x)f_{\theta_{j}}(x)}}.$$

That is, the relative probabilities that each component would generate the given gene conversion tract.

Then we update the parameters (M-step): and


$${\hat{\alpha }}_{i}^{\left(k+1\right)}=\sum_{t\in T}\frac{w_{i}^{t}}{\left|T\right|},$$

(8) $$\theta_{i}^{\left(k+1\right)}=\text{arg}\underset{\theta }{\max}\sum_{t\in T}w_{i}^{t}\;\text{log}(Pr(O=o_{t}|\theta ,S\neq Ø))=\text{arg}\underset{\theta }{\max}\sum_{t\in T}w_{i}^{t}\;\text{log}\;\left(\frac{\sum_{x=1}^{\infty }T_{t}(x)\cdot f_{\theta }(x)}{\sum_{x=1}^{\infty }D(x)\cdot f_{\theta }(x)}\right).$$

The log-likelihood will improve by at least the difference of the values of the target function at $\theta_{i}^{\left(k+1\right)}$ and $\theta_{i}^{\left(k\right)}$ for each component *i* (Dempster *et al.* 1977).

This guarantees a monotonic increase in the total log-likelihood. We repeat this process until the parameters have converged (by default the Euclidean norm of the relative changes in all parameters is less $10^{-7}$). We use scipy.optimize to find a value of *θ* that maximizes the sum.

Additional information on how we approximate the infinite sums is provided in the Supplementary Information.

### 2.4 Simulated data

We used the marker set from Jónsson *et al.* (2017) to construct a human-like set of heterozygous markers to use as an informative marker set. We used allele frequencies to determine the probability of each marker to be included in the heterozygous marker set. The mean and median distance between consecutive markers in the set was 1465 and 754, respectively. We tested our method in two experiments, E1 and E2, using simulated gene conversion tract datasets from NCO events drawn from negative binomial length distributions placed uniformly in the genome. The gene-converted status of each heterozygous marker overlapping the simulated NCO event was determined using a Bernoulli trial with the penetrance as the probability of each marker becoming gene converted. In Experiment E1, we simulated gene conversion tracts for 63 different parameter combinations, having mean 100, 300, and 1000 bp; 7 different values for the variance; and 0.5, 0.75, and 1.0 as the value for the penetrance—the proportion of heterozygous markers overlapping NCO events leading to gene conversions.

Since previous studies modelled the length distribution of NCO events with an exponential distribution, we reimplemented one of them, the method used by Li *et al.* (2019), for comparison. We also augmented that method to include the penetrance to see whether improvements on previous methods are due to the inclusion of penetrance or due to a more flexible model.

In Experiment E2, we simulated a mixture distribution by reusing two groups of datasets from E1, i.e. the ones with means 100 and 1000. For each pair of datasets from those two groups, we created a mixture with an equal probability of producing gene conversion tracts from both distributions. This results in 147 different parameter combinations (three different values of penetrance and seven different values for the variances of each of the two distributions having means 100 and 1000).

Each experiment was repeated 200 times to get a distribution for the inferred mean tract length and inferred number of NCO events.

Using the data from the first 20 repeats of E2 and each parameter combination (a total of 2940 datasets), we show that confidence intervals for the distribution mean and the total number of NCO events can be estimated by running NCOurd on resampled gene conversion tracts.

For each such dataset, we create 200 bootstrap samples of gene conversion tracts and run NCOurd on each sample. Thus, we get 200 estimates of the mean length and the number of NCO events from which we compute 95% confidence intervals.

Finally, we count how often the actual mean falls within the computed confidence intervals. This should happen in approximately 95% of the cases for all parameter combinations.

We calculated the confidence intervals for the published datasets using this bootstrapping method.

### 2.5 Determining the number of mixture components

Choosing the appropriate number of mixture components for the model is important. If too few components are used, the estimated length distribution of NCO events can lack some features of the true length distribution. More mixture components will always produce higher likelihood estimates, but too many will lead to overfitting the data.

We use a likelihood ratio test (Neyman and Pearson 1933) to determine the appropriate number of mixture components. A model with the length distribution of NCO events being a mixture of *n* negative binomial distributions is nested in a model with the length distribution of NCO events being a mixture of *n *+* *1 negative binomial distributions. The larger model has three additional degrees of freedom (an extra mixture weight and two extra parameters for the negative binomial distribution). Let $L_{n}$ and $L_{n+1}$ be the maximum likelihood with models having *n* and *n *+* *1 components, respectively. The null hypothesis is that the data obey the distribution of the model with *n* components. Given the null hypothesis, we have that $-2\;\text{log}(L_{n+1}/L_{n})$ approximates a $\chi^{2}$ random variable with three degrees of freedom (Wilks 1938), which in turn can be used to obtain a *P*-value.

Similarly, a model with the length distribution of NCO events as a geometric distribution is nested in a model with the length distribution of NCO events as negative binomial distributions. The larger model has one additional degree of freedom (an extra parameter). Let $L_{G}$ and $L_{N}$ be maximum-likelihood estimates with geometric and negative binomial distribution models, respectively. Assuming the null hypothesis that geometric distribution is the correct choice of a model, then $-2\;\text{log}(L_{N}/L_{G})$ approximates a $\chi^{2}$ random variable with one degree of freedom, which can be used to obtain a *P*-value.

To determine the number of mixture components to use, we start with one component and repeatedly add a component while the likelihood ratio gives a significant *P*-value less than .05.

## 3 Results

For the E1 experiments, we evaluated how well the two methods inferred the mean length and the number of NCOs for 1000 gene conversion tracts from a single distribution (one mixture component). We did this for each of the simulated datasets, repeating each experiment 200 times to get a distribution for the inferred values.

**Figure 2.**
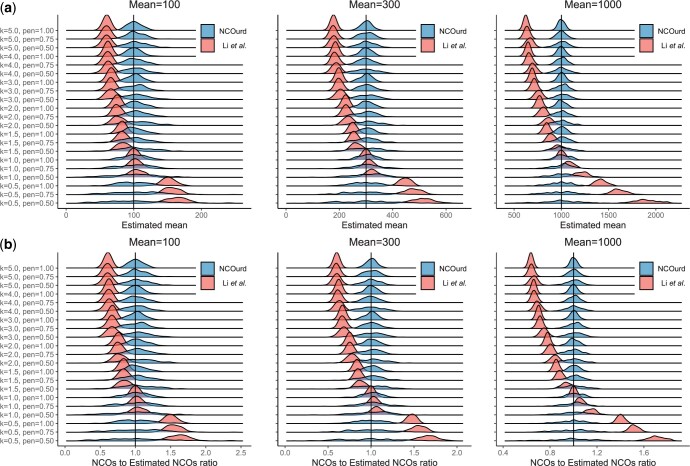
Results for simulated data. (a) Distribution of estimated mean NCO event length. (b) Distribution of the number of NCOs. NCOurd in blue and the method from Li *et al.* in red. Each experiment consists of 1000 simulated tracts from the given negative binomial distribution and was repeated 200 times to obtain a distribution. The penetrance for the datasets is ‘pen’ and the variance is ${\text{mean}}^{2}\cdot k$.

For NCOurd, the median of the inferred values for the mean length and number of NCOs agrees with their true values in all cases, while the Li *et al.* method is biased away from the mean when the distribution deviates from an exponential distribution (Fig. 2a and b). Supplementary Table S2 shows the downward bias of the mean estimate when the penetrance is omitted in the Li *et al.* method. Supplementary Fig. S2a shows how the variance of the mean estimation decreases with an increased number of tracts; the variance is inversely proportional to the number of tracts (Supplementary Table S3). As the standard deviation is the square root of the variance, a 4-fold number of input gene conversion tracts is needed to halve the standard error of the estimated mean NCO event length.

Next, for the E2 experiments, we evaluated how well NCOurd inferred mean NCO event length and the number of NCO events for 2000 gene conversion tracts from a mixture of two distributions with means 100 and 1000, using 1000 tracts from each distribution. For a mixture of two simulated datasets, the median values for mean length and the number of NCOs are close to the true values, but the variance is large if the small distribution has small *k* and the penetrance is low (Supplementary Fig. S2b and c).

For the simulated mixture distributions, we bootstrapped the input gene conversion tracts to estimate 95% confidence intervals. The mean NCO event length used for the simulations was within the estimated confidence interval 2784 out of 2940 or 94.7% [95% confidence interval (CI), 93.8%–95.4%], and the number of NCO events in the simulations was within the estimated confidence interval 2786 out of 2940 or 94.8% (95% CI, 94.0%–95.5%). This shows that bootstrapping the input gene conversion tracts accurately estimates the CIs.

Finally, we employed NCOurd on two publicly available gene conversion datasets.

The first dataset is from Li *et al.* (2019) consisting of 1575 gene conversion tracts from cross-breeding two highly divergent mouse strains. The mouse dataset contains a very dense set of markers with mean and median distance between consecutive markers of 179 and 67, respectively. Each strain was almost fully inbred, making the task of identifying gene conversions that accumulated over a few generations relatively straightforward since there were only three possible haplotype pairs: homozygous for either strain and heterozygous. On average, the set of heterozygous markers is half of the genome for each mouse. Penetrance was estimated as 0.9, computed as the fraction of gene-converted markers in all gene conversion tracts, excluding boundary markers (first and last marker of each tract).

For the inbred mouse data, we used a likelihood ratio test to determine the number of components for the mixture. We reject the null hypotheses that a single geometric distribution fits the data as well as a single negative binomial distribution with *P*-value $1.7\cdot 10^{-23}$, and that a single negative binomial distribution fits the data as well as a mixture of two negative binomial distributions with *P*-value $5.1\cdot 10^{-28}$. We cannot, however, reject the null hypothesis that a mixture of two negative binomial distributions fits the data as well as a mixture of three negative binomial distributions, *P*-value 0.36 (Supplementary Table S1). Out of the three models, the single geometric distribution is most comparable to the methodology of Li *et al.* (2019).

Using two negative binomial distributions gives an estimate of mean NCO event length of 42 bp (95% CI, 24–48) and an estimate of the number of NCO events repaired with the homologous chromosome per meiosis of 172.4 (95% CI, 152.2–290.1) (Table 1). Combining the estimated number of NCO events with the estimated number of crossovers gives 199.3 (95% CI, 179.0–316.9.1) DSBs repaired with the homologous chromosome using NCOurd. Li *et al.* estimate the average number of NCO events as 274 and the number of DSBs as 300.5. It is worth noting that Li *et al.* (2019) proposed a strand-aware model to obtain this estimate, while under a standard strand-unaware model, their estimate was 465.

**Table 1.** Estimated mean length of NCO events and the number of NCO events per meiosis using a single negative binomial distribution and a mixture of two negative binomial distributions for the datasets from Li *et al.* (2019) and Halldorsson *et al.* (2016).

	Distributions	Mean	NCOs	*P*-value
Mice	1	18.0 (12.5–26.0)	396 (273–552)	$1.7\cdot 10^{-23}$
Li *et al.* (2019)	2	41.8 (24.3–47.9)	172 (152–290)	$5.1\cdot 10^{-28}$
Human maternal	1	10.6 (7.3–17.5)	24445 (16202–31 603)	$4.3\cdot 10^{-46}$
Halldorsson *et al.* (2016)	2	41.9 (16.4–2925)	6858 (108–16 692)	0.0035
Human paternal	1	2.6 (1.65–11.9)	41994 (8676–51 537)	$5.3\cdot 10^{-48}$
Halldorsson *et al.* (2016)	2	177 (61.0–389)	791 (330–2098)	$4.1\cdot 10^{-13}$

*Notes*: Estimates are obtained with NCOurd with 95% CIs shown in parentheses. *P*-values were obtained with likelihood ratio tests, comparing single negative binomial distributions against single geometric distributions and mixtures of two negative binomial distributions against single negative binomial distributions.

The authors estimated the mean NCO event length separately depending on the controlling PRDM9 allele and inferred the mean length of NCO events as 30 and 41 bp for events controlled by the *Cast* and *Hum* PRDM9 alleles, respectively. Applying NCOurd, we estimate the mean NCO event lengths 17 (95% CI, 9.5–47) and 48 (95% CI, 40–57) for *Cast* and *Hum* controlled events, respectively, suggesting we lack power to determine whether their means differ.

A detailed description of the method used to estimate the number of NCO events can be found in the Supplementary Information, together with a comparison with the method used in Li *et al.* (2019).

The second dataset is from Halldorsson *et al.* (2016) consisting of 504 gene conversion tracts found and verified in large sequenced families. The dataset contained 257 paternal and 247 maternal gene conversion tracts. As gene conversions are known to behave differently depending on the sex of the transmitting parent (Halldorsson *et al.* 2016) we ran NCOurd separately on the paternal and maternal gene conversion tracts. Two maternal gene conversion tracts were excluded since they spanned more than 100 000 bp. Penetrance was estimated 0.52 using the method described above for the mouse dataset, and the mean and median distance between consecutive informative markers in the dataset is 2745 and 561, respectively.

Using a likelihood ratio test on paternal and maternal gene conversion tracts, we rejected the null hypothesis that a single negative binomial distribution fits the data as well as a mixture of two negative binomial distributions with *P*-values $4.1\cdot 10^{-13}$ and 0.0035, respectively. But we could not reject the null hypothesis that a mixture of two negative binomial distributions fits the data as well as a mixture of three negative binomial distributions, *P*-values 0.867 and 0.256, respectively.

We estimate the mean paternal and maternal NCO event length as 177 bp (95% CI, 61.0–389) and 41.9 bp (95% CI, 16.4–2925), respectively, and the number of paternal and maternal NCO events as 791 (95% CI, 330–2098) and 6858 (95% CI, 108–16 692), respectively (Table 1).

For NCOurd, the confidence intervals for the mean length and the number of maternal NCO events span two orders of magnitudes suggesting that a much larger dataset is needed for an accurate estimate. The confidence intervals for the mean length and the number of paternal NCO events are more reasonable. Using the method from Li *et al.* (2019), we get the estimate for mean NCO event length as 2506 (95% CI, 840–5415) and 21014 (95% CI, 9642–35 100) for paternal and maternal gene conversion tracts, respectively. Due to the presence of very long gene conversion tracts, the method from Li *et al.* (2019) fails to produce plausible mean estimates.

## 4 Discussion

We modelled the length distribution of NCO events and implemented NCOurd—an EM algorithm to infer NCO event lengths as a mixture of negative binomial distributions that best fit a gene conversion tract dataset. We have shown by simulations that the NCOurd accurately infers the mean length of NCO events and the number of NCO events occurring during meiosis and can be used to estimate confidence intervals for the inferred values. We demonstrated NCOurd on publicly available mouse and human gene conversion datasets to infer the mean lengths and the number of events for the underlying NCOs creating these gene conversions. For both datasets, a mixture of two negative binomial distributions fits the data significantly better than a single distribution. We presume that this is due to NCO events arising via multiple pathways, such as the synthesis dependent strand annealing and double Holliday junction pathways (Supplementary Fig. S1).

The main limitation of NCOurd is that the method requires more extensive data than is generally made available in gene conversion studies and that the method could be biased if the gene conversion process deviates drastically from the model assumptions. However, future researchers can easily create the extra data needed, the informative marker sets, and informative markers flanking each gene conversion tract. Most of the model assumptions are used to calculate the input for the EM algorithm, the detection function, and the tract functions. The strongest modelling assumptions are that the penetrance is fixed (i.e. all informative markers contained in any NCO event have the same probability of becoming gene converted) and NCO events are uniformly distributed in the genome. In the Supplementary Information, we discuss how the model assumptions can be relaxed.

As NCOs are understudied compared with the other contributors to genetic diversity, mutations, and crossovers, we hope this method will help us gain a better insight into NCOs and the meiotic recombination process in general. The true length distributions of NCO events will likely remain unknown for some time, but NCOurd can shed light on some of the features of the distribution, difference between the sexes, and eventually further our understanding of processes in oocytes during meiotic arrest leading to an increased number of crossovers and gene conversions.

## Supplementary Material

btad485_Supplementary_Data

## Data Availability

The datasets were derived from sources in the public domain: Li R, Bitoun E, Altemose N *et al.* A high-resolution map of non-crossover events reveals impacts of genetic diversity on mammalian meiotic recombination. Nat Commun 2019;10:3900–15. https://www.nature.com/articles/s41467-019-11675-y#Sec24. Halldorsson BV, Hardarson MT, Kehr B *et al.* The rate of meiotic gene conversion varies by sex and age. Nat Genet 2016;48:1377–84. https://www.nature.com/articles/ng.3669#Sec25. Jónsson H, Sulem P, Kehr B et al. Data descriptor: whole genome characterization of sequence diversity of 15,220 icelanders. Sci Data 2017;4:170115. https://www.ebi.ac.uk/ena/browser/view/PRJEB15197.
