# Supplementary material for: NCOurd: modelling length distributions of NCO events and gene conversion tracts
Source: Bioinformatics. 2023 Aug 3;39(8):btad485. doi: 10.1093/bioinformatics/btad485 (PMC10421967; doi:10.1093/bioinformatics/btad485)
Supplement: btad485_Supplementary_Data [file btad485_supplementary_data.pdf]

NCOurd: Modelling length distributions of NCO events and gene conversion tracts

Marteinn T. Hardarson[1,2], Gunnar Palsson[1], and Bjarni V. Halldorsson[1,2]

[1]deCODE genetics, Amgen, Sturlugata 8, Reykjavik, Iceland
[2]School of Technology, Reykjavik University, Reykjavik, Iceland.

Supplementary figures

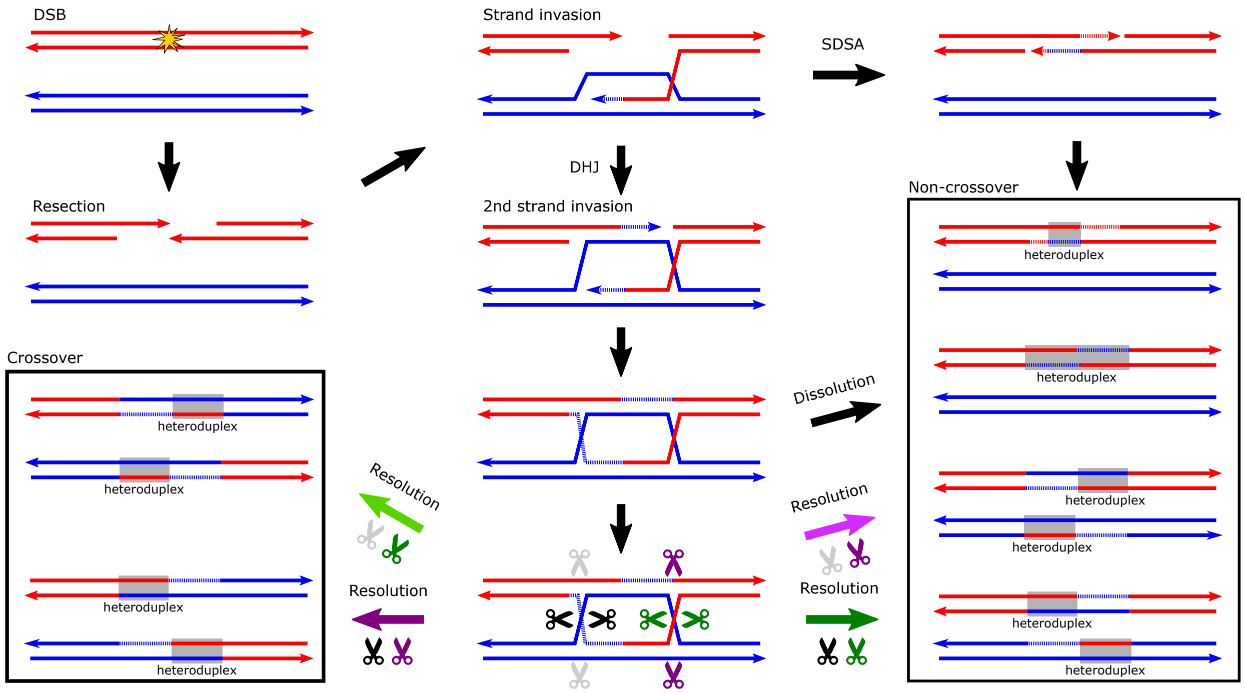

Figure S1: A DSB (yellow star) is induced on one chromosome (red) and is subsequently repaired using the homologous chromosome (blue) as a template. The lagging strands facing the DSB are resected. The overhang leading strands invade the homologous chromosome and DNA is synthesised (dotted lines) to bridge the DSB. If one strand invades the synthesis dependent strand annealing (SDSA) pathway is used, leading to potential gene conversions. If both strands invade the double Holliday junction (DHJ) pathway is used, leading to a potential gene conversion if the DHJ is dissolved and to a either gene conversion or a crossover if it is resolved (scissors). Heteroduplex segments (grey boxes) harbour mismatching base pairs at heterozygous markers and can become either allele in the offspring. The penetrance, the proportion of heterozygous markers overlapping NCO events leading to gene conversions, is governed by mismatched base pairs in heteroduplex segments resolved to gene conversion or to the background allele.

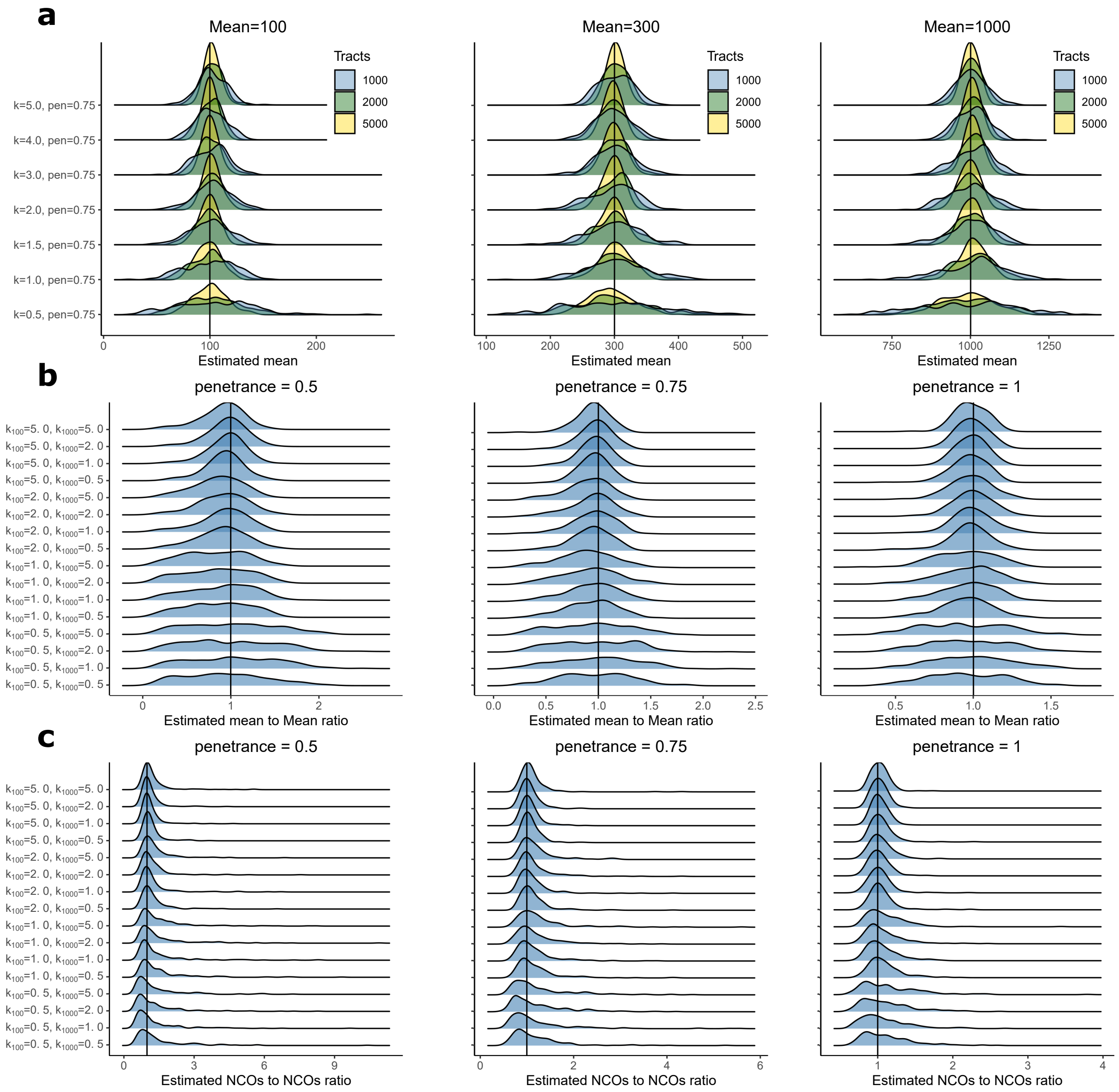

Figure S2: (a) Distribution of estimated means narrows around the true mean with a larger number of gene conversion tracts. **(b)** Distribution of estimated mean. **(c)** Distribution the estimated number of NCOs. The estimates are obtained by running NCOurd for 1000 tracts with mean 100 and 1000 tracts with mean 1000. Each experiment is repeated 200 times to estimate the distribution. The penetrance for the datasets is pen and the variance is mean$^2 \cdot k$ where indicated. In **(b)** and **(c)** k_{100} and k_{1000} are the values for k in the distributions with means 100 and 1000, respectively.

Supplementary tables

Distribution	NCO %	GC %	Mean	k
One geometric	100%	100%	51	1.0
One nbinom	100%	100%	18	0.19
Two nbinom	99.1%	97.1%	39	1.19
	0.9%	2.9%	360	0.79

Table S1: The result of NCOurd for a geometric distribution, a negative binomial distribution, and a mixture of two negative binomial distributions. Fraction of NCOs and gene conversion (GC) tracts belonging to each group, the estimated mean and parameter k are shown.

Mean	50% penetrance	75% penetrance	90% penetrance
100	42.4 (31.5,54.1)	69.4 (55.7,83.8)	88.1 (75.3,103.3)
300	140.5 (117.5,162.8)	216.2 (189.2,244.0)	265.1 (233.1,295.6)
1000	445.1 (397.4,493.2)	686.5 (629.8,744.3)	865.5 (789.6,937.1)

Table S2: Estimated mean compared to the true mean for the method from Li et al. (2019) when the penetrance is not used. Shown for geometric distributions with different means and different penetrance. Mean results and 95% confidence intervals for 1000 tracts from each sampled data set. The tracts are sampled 200 times.

Exponent	(95% confidence interval)
-1.041	(-1.059, -1.023)

Table S3: The variance of the estimated mean with NCOurd is inversely proportional to number of tracts. Results from the linear model $\log(\text{var}) \sim \log(n) + \text{cat}$ is shown, giving $\text{var} \sim n^{-1.041}$. Where n is the number of tracts, cat is categorical variable for each set of simulation parameters (penetrance, mean, k), and var is the variance calculated with bootstrapping from 200 repeated experiments for each category and number of tracts.

	Short	Long	Total
Mean NCO length	39 (4.5-45)	272 (38-958)	42 (24-48)
GC tracts %	96.7% (4.33%-99.1%)	3.3% (0.9%-0.95.7%)	100%
NCO events %	98.8% (18.4%-99.8%)	1.2% (0.2%-81.6%)	100%
NCOs detected %	7.52% (1.29%-17.1%)	20.03% (11.2%-82.5%)	7.67% (9.13%-17.4%)
All NCOs estimated	20266 (4289-25394)	256.4 (34.6-19933)	20522 (18100-34498)
NCOs per meiosis	170.3 (36.1-213.5)	2.15 (0.29-167.5)	172.5 (152.1-290.0)

Table S4: Results for two negative binomial distribution are shown with 95% confidence intervals are shown in parentheses: Mean length of NCO events; fractions of gene conversion tracts and NCOs belonging to the short and the long distributions; Expected number of NCO tracts detected as gene conversion tracts; Expected number of NCO events in all the sequenced genomes; and the total number of NCOs (repaired with the homologous chromosome) expected to occur each meiosis.

Supplementary methods

Computational approximations

To make NCOurd more efficient we use some approximations when calculating the log-likelihood function (logarithm applied to (6)) and membership weights (7) and when we updating the parameters $\boldsymbol{\theta}$ (8). All these equations contain:

$$g(x) = \frac{\sum_{x=1}^{\infty} T_t(x) \cdot f_\theta(x)}{\sum_{x=1}^{\infty} D(x) \cdot f_\theta(x)}.$$

We can approximate the infinite sums by the partial sums up to $x = K$ for some large K. Since NCO events are expected to be relatively short on average much shorter than 100,000 base pairs we use $K = 100,000$ and get:

$$g(x) \approx \frac{\sum_{x=1}^{K} T_t(x) \cdot f_\theta(x)}{\sum_{x=1}^{K} D(x) \cdot f_\theta(x)}.$$

To further simplify calculations we only evaluate the terms in the sums on a grid consisting of every integer from 1 up to 1,000, every 10th integer for 1,000 up to 10,000 and every 100th integer from 10,000 to 100,000. The value of $T_t(x) \cdot f_\theta(x)$ and $D(x) \cdot f_\theta(x)$ at non-grid point is the linear interpolation of the values at the closest smaller and larger grid points. The values of the functions $D(x), T_t(x)$ and $f_\theta(x)$ change slowly for large x and linear interpolation allows us to reduce computation by computing the functions on a grid. Summations involving these functions are then computed using the appropriate weights. The weights are chosen so that the approximation is exact for piecewise linear functions between grid points. Let $h(x)$ be the terms in the sum rewritten as

$$\sum_{x=1}^{K} h(x) \approx \sum_{x \in grid} c_x h(x),$$

where

$$c_x = \begin{cases} 1 & \text{if } 1 \leq x \leq 999 \\ 5.5 & \text{if } x = 1,000 \\ 10 & \text{if } 1,001 \leq x \leq 9,999 \\ 55 & \text{if } x = 10,000 \\ 100 & \text{if } 10,001 \leq x \leq 99,999 \\ 50.5 & \text{if } x = 100,000. \end{cases}$$

When we updating the parameters $\boldsymbol{\theta}$ (8) we also use the following algebraic manipulation:

$$\begin{aligned} &\sum_{t \in \mathcal{T}} w_i^t \log \left(\frac{\sum_{x=1}^{\infty} T_t(x) \cdot f_\theta(x)}{\sum_{x=1}^{\infty} D(x) \cdot f_\theta(x)} \right) \\ &= \sum_{t \in \mathcal{T}} w_i^t \log \left(\sum_{x=1}^{\infty} T_t(x) \cdot f_\theta(x) \right) \\ &- \left(\sum_{t \in \mathcal{T}} w_i^t \right) \cdot \log \left(\sum_{x=1}^{\infty} D(x) \cdot f_\theta(x) \right) \end{aligned}$$

Mixture of Distributions

Denote $f(x)$ and $\widehat{f}(x)$ be the probability mass functions (PMF) for the length distributions of NCO events and gene conversion producing NCO events ($S \neq \emptyset$), respectively. Using (3) we get

$$\widehat{f}(x) = \frac{f(x)D(x)}{\sum_{x=1}^{\infty} f(x)D(x)}.$$

Let $\mathcal{M}$ be the set of PMFs having positive integers as domain. We define the function

$$\mathcal{D} : \mathcal{M} \to \mathcal{M}, f \mapsto \widehat{f}.$$

If the length distribution of NCO events is a mixture of distributions

$$f(x) = \sum_{i=1}^{n} \alpha_i f_i(x),$$

then

$$\begin{aligned}
\widehat{f}(x) &= \frac{\sum_{i=1}^{n} \alpha_i f_i(x) D(x)}{\sum_{x=1}^{\infty} f(x) D(x)} \\
&= \sum_{i=1}^{n} \frac{\alpha_i}{\sum_{x=1}^{\infty} f(x) D(x)} \frac{\sum_{x=1}^{\infty} f_i(x) D(x)}{\sum_{x=1}^{\infty} f_i(x) D(x)} f_i(x) D(x) \\
&= \sum_{i=1}^{n} \alpha_i \frac{\sum_{x=1}^{\infty} f_i(x) D(x)}{\sum_{x=1}^{\infty} f(x) D(x)} \widehat{f}_i(x) \\
&= \sum_{i=1}^{n} \widehat{\alpha}_i \widehat{f}_i(x),
\end{aligned}$$

so

$$\mathcal{D}\left(\sum_{i=1}^{n} \alpha_i f_i\right) = \sum_{i=1}^{n} \widehat{\alpha}_i \mathcal{D}(f_i),$$

with

$$\widehat{\alpha}_i = \alpha_i \frac{\sum_{x=1}^{\infty} f_i(x) D(x)}{\sum_{x=1}^{\infty} f(x) D(x)}.$$

This shows that a length distribution of gene conversion producing NCO events is also a mixture of the distributions $\widehat{f}_1, \ldots, \widehat{f}_n$.

From (4) we also see that α_i is proportional to $\widehat{\alpha}_i / \left(\sum_{x=1}^{\infty} f_i(x) D(x)\right)$ and their sum is 1 so we can calculate α_i with

$$\alpha_i = \frac{\widehat{\alpha}_i / \left(\sum_{x=1}^{\infty} f_i(x) D(x)\right)}{\sum_{j=1}^{n} \widehat{\alpha}_j / \left(\sum_{x=1}^{\infty} f_j(x) D(x)\right)}.$$

The Penetrance

To estimate the detection function and the tract functions we need to consider the penetrance. For this purpose we assume that all informative markers contained within all NCO events for all parent-offspring pairs have the same probability p of being gene-converted. $Pr(s \in S) = p$ for all R and $s \in [M, M + L - 1] \cap I_R$. This assumption can be relaxed by allowing the penetrance to be a function of the length of the NCO event and write $p(x)$. In this case we would have $Pr(s \in S \mid L = x) = p(x)$ for all R and $s \in [M, M + x - 1] \cap I_R$.

To evaluate the penetrance of a real dataset we need to examine the markers within gene conversion tracts. The first and last markers within a gene conversion tract are always gene-converted by definition. However, other markers within the gene conversion tracts have a probability p of being gene-converted and the maximum likelihood estimate of the penetrance for these markers is just the fraction of such markers being gene-converted

$$p = \frac{\#geneConvertedInteriorMarkers}{\#allInteriorMarkers}.$$

Approximating the Detection Function

The detection function is $D(x) = Pr(S \neq \emptyset \mid L = x)$. This is the probability of observing an NCO event of length x. Calculating the detection function for all values of x is computationally expensive so we evaluate it for a few values of x and, for other values of x, we interpolate the value of D using one dimensional cubic interpolation. To evaluate D for a given x we walk along each chromosome placing an NCO event of length x at each position in the genome and use the penetrance to calculate the probability of the NCO event being observed as a gene conversion tract on the informative marker set. Our model assumes that each chromosomal position has the same probability, an assumption

that may not hold as NCO events cluster in hot-spots. This can be accounted for by using weights at each chromosomal position or by using only some regions of the genome. In both cases some additional data on NCO hot-spots and some decisions on how to determine the weights or regions are required. If the NCO event overlaps n informative markers, the probability of it becoming a gene conversion tract is

$$1 - (1-p)^n,$$

where p is the penetrance.

Then $D(x)$ is the sum of probabilities over all placements. This is done for each parent-offspring pair separately, and the final detection function is the average of the parent-offspring pairs' detection functions over all parent-offspring pairs. This assumes that a random NCO event has the same probability of occurring in any parent-offspring pair. Even though this assumption might not be fulfilled, we expect informative marker density to be roughly the same across all parent-offspring pairs. Alternatively, one can use a weighted average instead of these probabilities.

We tested different numbers of calculated values and we estimate that the function can be computed accurately by computing only a few values.

$$\begin{aligned} x = 1, \quad 10, \quad & 20, \quad 30, \quad \cdots \quad 100, \\ & 200, \quad 300, \quad \cdots \quad 1000, \\ & 2000, \quad 3000, \quad \cdots \quad 10000, \\ & 20000, \quad 30000, \quad \cdots \quad 100000. \end{aligned}$$

The detection function is used both as an input to the EM algorithm and to evaluate the number of NCO events afterwards (see "Estimating the Number of NCO Events"). For the inbred mouse data (Li *et al.* (2019)) we used an alternative method of calculating the detection function. As the weight for each random gene conversion event placement we used the number of times the midpoint of the random gene conversion tract for the placement is within 100Kb of a gene conversion tract from the data set.

Approximating the Tract Functions

For a tract $t \in \mathcal{T}$ we defined a tract function $T_t(x) = Pr(O = o_t \mid L = x)$. This is the probability of an NCO event of length x producing the gene conversion tract t. Since an NCO event can only cause gene-converted markers within its boundaries, it suffices to look only at placements of the NCO events containing the whole gene conversion tract. For a fixed NCO event containing the gene conversion tract, let the number of gene-converted and non-gene-converted informative markers within the NCO event be m and n, respectively. Let p be the penetrance. The probability of the NCO event producing the given gene conversion tract is $p^m(1-p)^n$. Then $T_t(x)$ is the sum of these probabilities over all possible placements of an NCO event of length x containing the gene conversion tract divided by the size of the genome (assuming uniform distribution of NCO event placements).

Note that multiplying a tract function with a constant leads to the same estimated NCO length distribution as it doesn't affect the parameters maximising the likelihood function equation (6). In fact, equations (7) and (8) give exactly the same results as well. As a consequence of this we do not need uniform distribution of NCO event placements for the whole genome but only locally around the gene conversion tract.

Furthermore, all potential NCO events containing a given gene conversion tract will contain all informative markers within the tract, and only the number of non-gene-converted markers outside of the tract differ. Therefore, for all values x, the function $T_t(x)$ is multiplied with the constant factor $p^m(1-p)^{n-k}$ where $n-k$ is the number of non-gene-converted informative markers within the tract. If an NCO event of length x containing the tract has k informative markers that are outside of the tract its contribution to $T_t(x)$ is

$$\frac{(1-p)^k}{|genome|}.$$

Let t be a gene conversion tract and let A_0 be the position of the left most gene-converted marker of t and A_i be the position of the i-th informative marker to the left of t. Similarly, let B_0 be the positions of the right most gene-converted marker of t and B_j be the position of the j-th informative marker to the right of t. Let l be the number of base pairs spanning the gene conversion tract and let $a_1, a_2, \ldots$ and $b_1, b_2, \ldots$ be the distances to first, second, etc. informative markers preceding and following the gene conversion tract, respectively. then

$$\begin{aligned} l &= B_0 - A_0 + 1, \\ a_i &= A_0 - A_i, \\ b_j &= B_j - B_0. \end{aligned}$$

If t contains only a single gene-converted marker we have $l = 1$. Non-gene-converted informative markers are represented with $\circ$ and gene-converted informative markers with $\bullet$ in the following diagram:

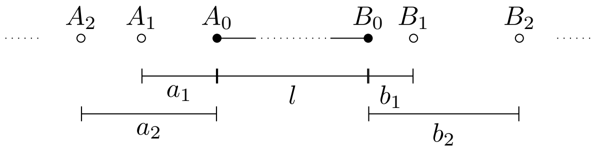

Let $a_0 = b_0 = 0$ and

$$\begin{aligned} r_{i,j} &= l + a_i + b_j, \\ s_{i,j} &= \min(a_{i+1} - a_i, b_{j+1} - b_j), \\ t_{i,j} &= \max(a_{i+1} - a_i, b_{j+1} - b_j). \end{aligned}$$

To count the number of possibilities to place an NCO event of length $x + r_{i,j}$ and having its left endpoint between A_i and A_{i-1} and its right endpoint between B_j and B_{j-i} we define the function

$$f_{i,j}(x) = \begin{cases} 0 & \text{if } x \leq 0 \\ x & \text{if } 0 \leq x \leq s_{i,j} \\ s_{i,j} & \text{if } s_{i,j} \leq x \leq t_{i,j} \\ s_{i,j} + t_{i,j} - x & \text{if } t_{i,j} \leq x \leq s_{i,j} + t_{i,j} \\ 0 & \text{if } s_{i,j} + t_{i,j} \leq x \end{cases}$$

having the graph:

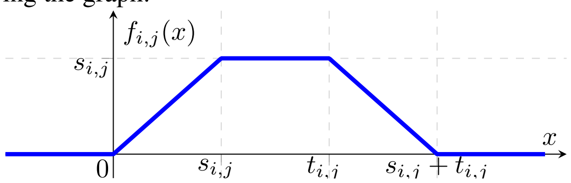

Then we have

$$T_t(x) = \frac{1}{|genome|} \sum_{i=0}^{\infty} \sum_{j=0}^{\infty} (1-p)^{i+j} f_{i,j}(x - r_{i,j})$$

where each term in the double sum corresponds to all NCO events containing t and the i informative markers preceding and the j informative markers following it. In practice $p \geq 0.5$ so because of the factor $(1-p)^{i+j}$ we only need to calculate the terms for $i, j \leq 20$.

Estimating the Number of NCO Events

Having determined the parameters for each component of the mixture of the distribution of NCO events, we can evaluate the probability of an event from a component i, having parameters θ_i, generating a gene conversion tract.

$$\begin{aligned} Pr(S \neq \emptyset \mid \theta_i) &= \sum_{x=1}^{K} Pr(S \neq \emptyset \mid L_{\theta_i} = x) \cdot Pr(L_{\theta_i} = x) \\ &= \sum_{x=1}^{K} D(x) \cdot Pr(L_{\theta_i} = x). \end{aligned}$$

If the expected number of gene conversion tracts from the component is $\widehat{E}_{\theta_i}$ then the expected number of NCO events from the component is

$$E_\theta = \frac{\widehat{E}_{\theta_i}}{Pr(S \neq \emptyset \mid \theta_i)}.$$

The component weight for the i-th component $\widehat{\alpha}_i$ is obtained from the EM algorithm and gives

$$\widehat{E}_{\theta_i} = \widehat{\alpha}_i \cdot |\mathcal{T}|.$$

Simulated Gene Conversion Tracts

We used the marker set defined in Jónsson *et al.* (2017) assuming a study design where informative markers are all the markers where the transmitting and non-transmitting parents are heterozygous and homozygous, respectively. Allele frequencies were used to determine the probability of a marker being informative and then each marker's informative status was determined via Bernoulli trials.

We simulated 63 sets of NCO events following negative binomial distribution. For each NCO event, we do Bernoulli trials with the penetrance as probability to determine which informative markers became gene-converted. The NCO event length distributions had mean m, variance m^2/k and penetrance p for all combinations of

$$m = 100, 300, 1000$$
$$k = 0.5, 1, 1.5, 2, 3, 4, 5$$
$$p = 0.5, 0.75, 1.$$

The value k corresponds to the shape parameter in the gamma distribution having the same mean and variance as the negative binomial distribution.

We then inferred the maximum likelihood distribution for 1000 gene conversion tracts separately from each tract set with two methods NCOurd and for comparison we re-implemented the method used in Li *et al.* (2019) adding the possibility of inputting or inferring penetrance. The only difference to Li *et al.* (2019) is that their formula (4) becomes:

$$Pr(\text{SNP nearby converted}) = Pr(\text{in}) = p \cdot e^{\lambda d}$$

where we introduce the penetrance factor p. Each experiment was repeated 200 times to obtain a distribution of the estimated means and the estimated number of NCO events. As NCOurd needs to be provided the penetrance, the method from Li *et al.* was also run with the correct penetrance provided, as well as with 100% penetrance to illustrate the importance of including the penetrance while modelling gene conversions.

Inbred Mice

To run NCOurd on the data from Li *et al.* (2019) we must estimate the penetrance in order to calculate the detection and tract functions. Out of the 178 markers within gene conversion tracts excluding the boundary markers, 161 are gene-converted, giving 0.90 penetrance.

Calculating an exact detection function for each meiosis is impossible since the gene conversions are identified in genotyped mice but were generated when their non-genotyped ancestors produced germ cells. We can however estimate an average detection function across all meioses by halving the detection function for a mouse that is heterozygous on the whole genome. This works since at any locus each ancestral mouse is heterozygous with 50% probability.

To calculate the tract functions we know that every gene conversion tract was created during meiosis in an ancestor heterozygous at the locus. As crossovers are unlikely to occur in a window of size 100Kb, we assume that every marker in the window is heterozygous.

We ran NCOurd for a one geometric and one, two, and three negative binomial distributions and used a likelihood ratio test for the choice of a model and concluded that two negative binomial distributions best fit the data. If NCOs follow the estimated mixture of two negative binomial distributions the expected fraction of NCOs leading to gene conversion tracts is 7.67% leading to an estimate of 20,523 NCOs which occurred in 476 meiosis. This is however only 1/4 of the actual NCO events as only one of the four products of each meiosis is passed to the child.

We estimate the number of NCOs per meiosis to be 172.4 (95% CI 152.2-290.1) using the formula:

$$E[T] = \frac{K}{4} \cdot M \cdot \text{DetFrac}$$

where T is the number of gene conversion tracts, K is the number of NCO events per meiosis, M is the number of meiosis and DetFrac is the fraction of NCOs leading to gene conversion tracts (Table S4). We also estimated NCOs per meiosis separately for the second (F2), fourth (F4) and fifth (F5) generation mice and obtained comparable estimates: 216.8 (95% CI 191.3-364.6), 159.6 (95% CI 140.9-268.5) and 184.6 (95% CI 162.9-310.5), respectively.

We can also estimate the number NCOs using the method in Li *et al.* (2019) using their formula:

$$E[N] = \frac{K}{4} \cdot M \cdot \text{Power} \cdot L \cdot D$$

where N is the number of gene-converted markers, K is the number of NCOs, M is the number of meiosis, Power the estimated probability of detecting a gene-converted marker, L is average length of an NCO event and D is the marker density near DSBs. The authors use the formula to estimate K from estimates of all the other variables. We get a different estimate for L but otherwise use their estimates for the other parameters and estimate the number of NCOs as $K = 197$.

The authors estimated on average 26.8 COs per meiosis leading to an estimate of the total number of DSBs repaired with the homologous chromosome being 199.3 and 223.8 using the two methods.

Finally, we used an alternative method to calculate the detection function using only regions consisting of gene conversion tracts extended 100Kb in both directions instead of the whole genome. Note that loci containing multiple gene conversion tracts contributed multiple times to the detection function. This method takes DSB hot-spots into account but favours marker dense regions where NCOs have a better chance of being observed as gene conversions.

We ran NCOurd using this detection function giving the mean NCO length 41.89 base pairs compared to 41.82 base pairs before and the number of NCOs per meiosis 152.2 compared to 172.4 before. The estimate of NCOs per meiosis can be lowered due to marker dense regions.
